# Assessing the impact of virtual workplaces on collaboration and learning

**DOI:** 10.3389/fpsyg.2025.1581029

**Published:** 2025-06-19

**Authors:** Irene Sánchez Rodríguez, Luca Bailo, Folco Panizza, Valerie A. A. van Es, Marzio Alessi, Monica Betta, Emiliano Ricciardi

**Affiliations:** ^1^IMT School for Advanced Studies Lucca, Molecular Mind Laboratory, Lucca, Italy; ^2^Neuroscience Lab, Intesa Sanpaolo Innovation Center SpA, Turin, Italy

**Keywords:** metaverse, virtual reality, social cognition, collaboration, learning, virtual workplaces

## Abstract

**Introduction:**

The shift toward remote work and digitization has driven the widespread adoption of virtual collaboration tools. While these technologies offer opportunities for enhanced remote interaction, they also present challenges related to communication, security, and social dynamics. Understanding how different digital environments impact collaboration and learning is crucial as workplaces evolve.

**Methods:**

This study investigated the impact of four workplace environments—physical presence, videoconferencing, non-immersive Metaverse, and immersive Metaverse—on task performance, cognitive engagement, ergonomics, and social dynamics. A total of 103 participants performed an “active” social decision-making task and a “passive” information retention task. Behavioral and electrophysiological data (EEG-based indices of concentration, fatigue, and relaxation) were collected to assess cognitive and emotional states.

**Results:**

Virtual environments supported collaboration comparably to physical presence. However, immersive environments (Metaverse VR+) were associated with reduced concentration and increased cognitive load, particularly during passive learning. Female participants exhibited higher attentional focus across conditions, and older participants outperformed younger ones in certain tasks. Ergonomic factors such as device comfort and ease of use significantly influenced concentration and relaxation.

**Discussion:**

While virtual platforms can replicate many aspects of physical presence, key differences persist in cognitive load, comfort, and engagement—especially in immersive settings. These findings highlight the potential and limitations of technologies like the Metaverse in supporting remote collaboration and learning, emphasizing the need for thoughtful design to reduce fatigue and enhance usability.

## 1 Introduction

The world is moving toward a more digital future. Following the COVID-19 pandemic, digital strategies have accelerated the adoption of remote work solutions in both the public and private sectors (Pushpa et al., 2024). This shift has created an unprecedented incentive for companies to digitize their operations, leading to the adoption of software technologies for online meetings, remote work, and e-Commerce during the pandemic (Dyba and Di Maria, 2024). Many companies transitioned to fully remote or hybrid work models, driven by the need to comply with social distancing measures and maintain business continuity (Kähkönen, 2023).

Although its long-lasting effects still need to be fully understood, remote work has been linked to lower costs and higher profit margins for companies. For instance, consulting projects carried out remotely have demonstrated substantial savings and improved profitability (Bender Maritan et al., 2024). In addition, remote work has been associated with increased employee satisfaction and productivity (Becchetti et al., 2024). This shift toward digital strategies and remote work is expected to have lasting effects, with online meetings and reduced business travel emerging as long-term outcomes of digitization. In addition, this movement toward remote working also impacted on environmental benefits, such as reduced carbon emissions and traffic congestion. For example, remote workers in Italy saved an average of 6 kg of CO_2_ per day by avoiding commutes (Fortuna et al., 2023).

In this context, in addition to more “classical” videoconferencing software (e.g., Microsoft Teams, Google Meet, Zoom, etc.), the metaverse and virtual reality (VR) have emerged as novel candidates for simulating situations of presence (e.g., meetings, interviews, etc.), primarily when people cannot share the same physical places. The metaverse is an evolving concept that merges the physical and digital worlds into a 3D virtual environment, allowing users to interact through avatars in real-time. This immersive integration improves virtual collaboration, meetings, and various work-related activities, creating a more engaging and efficient experience that blends elements of actual reality with digital virtuality (Cali et al., 2022; Kalra et al., 2023; Kritika, 2024; Mandala et al., 2023; Riva et al., 2021; Yaqob and Hafez, 2023).

While offering enhanced immersion compared to traditional videoconferencing, these new technologies present both promising opportunities and emerging challenges. On one hand, these platforms can potentially facilitate more dynamic collaboration and interaction in remote work settings by increasing the sense of presence (Bayro et al., 2022) or enhancing interaction and communication (Higuchi et al., 2015). On the other hand, several concerns have been identified, such as difficulties in communication, coordination, and establishing shared understanding within virtual project teams, which can negatively affect outcomes (Owens et al., 2011). Privacy and security risks, including data breaches and cyberattacks, are raising concerns in virtual environments (Ali et al., 2023). When discussing interactions in virtual environments, it is also essential to acknowledge that ethical and moral issues, such as harassment and discrimination, are significant social challenges that may be amplified. The heightened sense of presence and anonymity provided by avatars, combined with real-time interaction, can lead to more intense social dynamics than conventional online spaces (Massari et al., 2024; Cheng et al., 2017).

Beyond the social and technical implications, it is crucial to consider the physical and cognitive ergonomic impacts of virtual workplaces. The collection of behavioral and electrophysiological data plays a vital role in understanding these aspects, enabling real-time monitoring of brain and body responses, providing insights into how users respond to virtual experiences and how these technologies affect their cognitive performance and overall wellbeing (Doren et al., 2017; Eoh et al., 2005). Cognitive load—i.e., the mental effort required to process information (Sweller, 2011)—, can increase with prolonged use of immersive technologies, particularly for beginners. This added mental effort, often caused by external factors like complex interfaces or unfamiliar tasks, can hinder attention and learning (Poupard et al., 2024). However, cognitive fatigue, also known as “mental fatigue”—distinct from the cognitive load—refers to the mental exhaustion resulting from prolonged engagement with these environments (Karim et al., 2024). Even though VR may also be used for stress reduction interventions, the required engagement in activities conveyed through VR could lead to cognitive fatigue over prolonged use (Nath et al., 2023, 2024). In addition, wearing head-mounted displays (HMDs) can create physical discomfort (e.g., eye strain, cybersickness, erroneous body postures, etc.), contributing to fatigue and reduced concentration over time (Matar et al., 2022). These physical restrictions can be a significant hindrance to user experience.

Despite the rapid growth and popularity of these technologies, few studies have examined their impact in work contexts. The metaverse appears to enhance the creative performance of virtual teams by providing a collaborative and immersive space for idea generation and problem-solving (Lee, 2023); creating immersive and interactive work environments also appears to increase employee performance and job satisfaction (Harthy et al., 2023). This increased engagement may foster greater productivity and overall job fulfillment. In addition, the metaverse promotes work-life balance by enabling flexible remote working options that allow employees to better manage their personal and professional responsibilities (Harthy et al., 2023).

Altogether, these initial observations highlighted the necessity to explore how the evolving dynamics of workplace environments, ranging from traditional in-person settings to videoconferencing and even to cutting-edge virtual platforms, may shape the future of work. Our exploratory research protocol sought to understand how these novel digital tools may influence cognitive performance, comfort, and social dynamics by investigating behavioral and physiological responses across four common and possible workplace scenarios while performing work-related tasks: in-presence, videoconferencing using Microsoft Teams (Teams), the non-immersive metaverse interacting via computer (Metaverse VR-), and the immersive metaverse with Virtual Reality (Metaverse VR+) implemented via a Head Mounted Display. In addition to behavioral measurements, we also collected electrophysiological data to evaluate participants' levels of fatigue, concentration, and stress/relaxation. These indices provide a more nuanced understanding of how virtual environments impact users' cognitive and emotional states. This approach goes beyond simply comparing different work environments and explores how these digital platforms can influence productivity, wellbeing, and workplace dynamics. By incorporating these physiological insights, we could more accurately assess the true potential of virtual environments to not only serve as alternatives to traditional workspaces but also to transform them. Ultimately, this integration strengthens the study's findings and offers a valuable perspective on the effectiveness and impact of virtual platforms in professional settings.

Based on these premises, the present study explores the following research questions: (1) How do different digital workplace environments—ranging from traditional in-person settings to immersive virtual platforms—impact cognitive engagement, collaboration, and ergonomics during active and passive work-related tasks? (2) How do individual demographic factors, such as age and gender, moderate users' cognitive and emotional responses within these environments? To answer these questions, we adopted a mixed-method approach combining behavioral, self-report, and electrophysiological (EEG) data. The main contributions of this work lie in: (i) providing empirical evidence on the cognitive and ergonomic implications of immersive and non-immersive virtual learning and collaboration tools; (ii) identifying how specific user characteristics may influence engagement and wellbeing in digital work contexts; and (iii) informing future design principles for inclusive and cognitively sustainable virtual workplace environments.

## 2 Methods and materials

### 2.1 Participants

One hundred and forty-eight employees from a major Italian banking group took part in the study voluntarily. We considered all employees with normal or corrected auditory and visual capabilities (with corrective lenses or glasses if needed) and normal upper limb mobility eligible to participate in the study. Participants were recruited through targeted communications to specific organizational areas through an internal platform. Of the 148 participants who initially responded to the demographic questionnaire, 103 completed the experimental procedure. One participant identified as non-binary and was excluded from gender-based analyses, resulting in a sample of 102 for gender-related comparisons, having a gender-balanced sample, with 49% of participants being female.

#### 2.1.1 Demographic questions

The demographic questionnaire administered to participants included the following questions: age, gender (with options: Male, Female, Non-binary, Prefer not to answer), number of children (if applicable), highest level of education (with options: High School, Undergraduate degree, Graduate degree, Postgraduate degree), whether they work in the IT sector (Yes/No), and their experience with Virtual Reality (VR). The detailed questions regarding participants' experience with Virtual Reality (VR), including usage frequency, Metaverse engagement, and gaming habits, are provided in the Supplementary material S1. Participants were classified as “experts” or “non-experts” based on a scoring system, where responses to VR usage, Metaverse engagement, and gaming frequency were mapped to numerical values. Participants with a total score above a threshold of 6, and who also answered “Yes” to any of the binary experience questions (such as familiarity with avatars or participation in virtual events), were classified as “experts”. Details on the demographic questionnaire, including questions related to age, gender, education, and prior experience with virtual reality (VR), are provided in Table 1.

**Table 1:** Demographic characteristics of participants across the four experimental scenarios (Presence, Teams, Metaverse with VR, and Metaverse without VR).

**Variable**		**Presence**	**Teams**	**Metaverse VR**	**Metaverse No VR**	**Total**
Age	Mean (SD)	41.6 (10.2)	43.8 (9.6)	37.5 (8.2)	40.7 (13.1)	41.1 (10.6)
Gender	Male	58.6%	37.5%	42.9%	66.7%	50.5%
	Female	48.3%	62.5%	57.1%	33.3%	49.5%
VR familiarity	Expert	6.9%	4.0%	14.3%	16.0%	9.7%
	Non-Expert	93.1%	96.0%	85.7%	84.0%	90.3%
IT job	IT-related	58.1%	60.0%	57.1%	76.0%	62.1%
	Non-IT	41.9%	40.0%	42.9%	24.0%	37.9%

Values for age are reported as mean and standard deviation. Categorical variables are reported as percentages within each condition.

Due to technical issues (e.g., intermittent Wi-Fi connectivity), occasional incomplete data and instances of physiological devices not recording correctly (such as the Muse 2 experiencing interference during in-person sessions), the final analyses were conducted on a sample of 86 for the passive task and 85 for the active task. All participants were randomly assigned to one of the four scenarios (see 2.4 for details). No compensation was provided to participants for their involvement in the study. Recruitment took place in October 2023. All procedures were approved by the Joint Ethical Committee for Research of Scuola Normale Superiore and Scuola Superiore Sant'Anna (number 44/2023).

### 2.2 Study design

Our study employed a mixed-method design to explore how different work environments influence performance, comfort, and social interaction during work-related tasks. We conducted an ecological experiment where participants performed two tasks: an active task designed to assess decision-making and social interaction, and a passive task to evaluate attention, comprehension, and learning (described in Section 2.3). These tasks were performed in four distinct scenarios: “Presence,” “Teams” (with the camera on), Metaverse VR-, and Metaverse VR+ (see 2.4 for details). This approach allowed us to compare the quantitative data from physiological and behavioral measures and the qualitative feedback of participants about ergonomics and subjective experience. The task order was counterbalanced in each experimental session so that the results obtained were not influenced by the order in which the tasks were presented. Participants completed the study in only one workplace scenario following a between-subject design. This avoided cross-scenario influences within the same session.

#### 2.2.1 Procedures

Upon arrival at the research site, participants met at a designated check-in point, where researchers welcomed and explained to them the general procedures. Each session consisted of four participants that were then escorted by the experimenters to their designated experimental environments and lasted approximately 60 min. For the Presence condition, participants were brought together to a shared room for the passive task, or divided into pairs and led to separate rooms for the active task, as detailed in Section 2.4.1. In all other conditions (Teams, Metaverse VR-, and Metaverse VR+), the four participants were separated into four individual rooms, where they remained for the entire session.

Each task was preceded by a brief setup phase during which participants were fitted with a MUSE 2 EEG headband and, when requested, the Oculus Quest 2 headset. EEG data were recorded continuously throughout the execution of each task. Following task completion, participants removed the VR and EEG wearable devices if deemed necessary, and completed post-task self-report questionnaires using a laptop. EEG was not recorded during this phase. Participants were allowed a short break while adjusting their equipment for the second task. At the end of the second task and the associated questionnaires, researchers debriefed the participants and provided a brief explanation of the study's goals and the nature of the collected measures.

### 2.3 Tasks

#### 2.3.1 Active task: Prisoner's Dilemma

**Figure 1:**
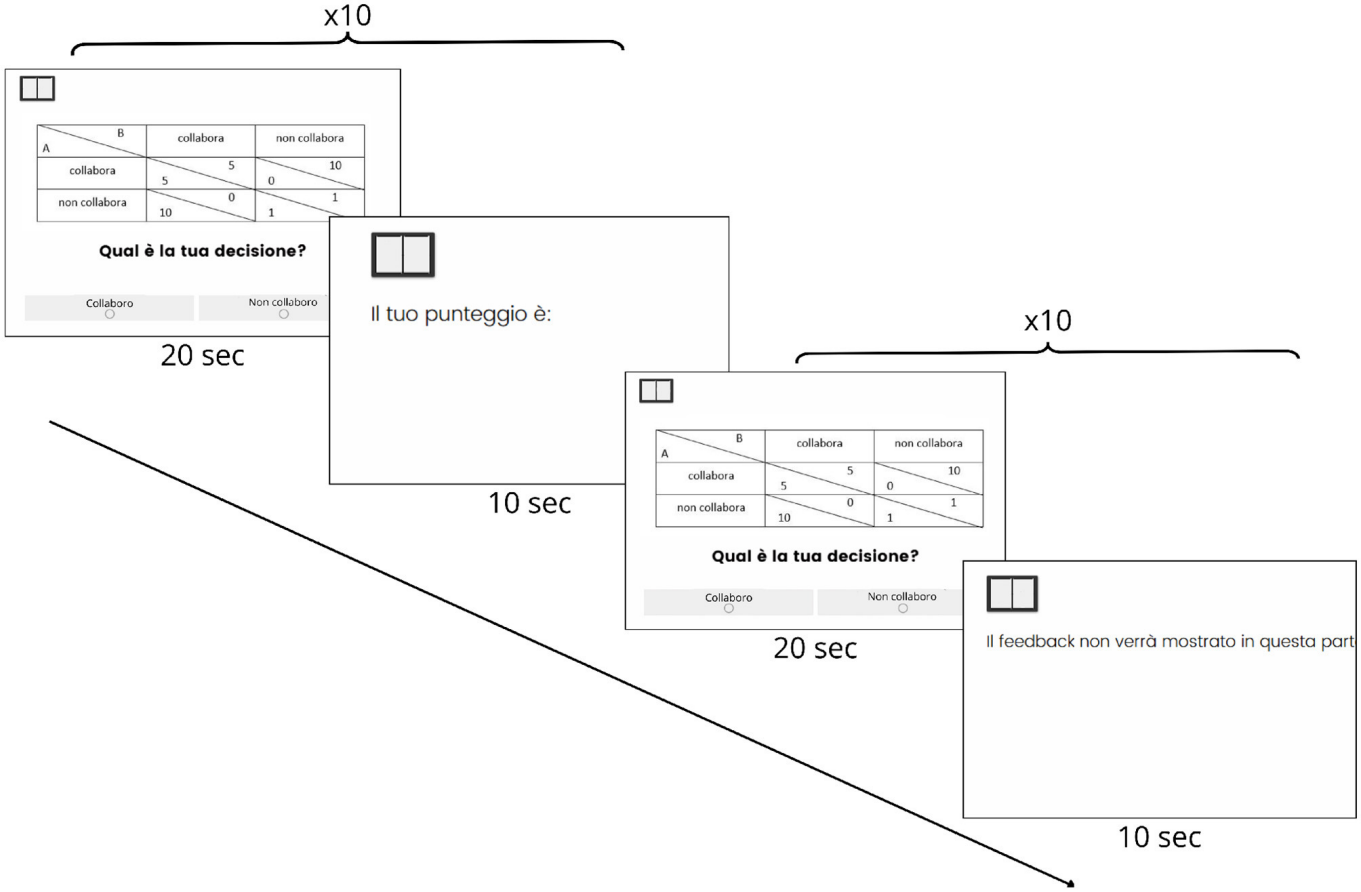
Actual footage of the experiment flow. The experiment was conducted in Italian. In each trial, participants are presented with the question “Qual è la tua decisione?” (“What is your decision?”) and are asked to choose between two options: “Collabora” (“Cooperate”) or “Non collabora” (“Do not cooperate”). After each choice, participants receive feedback on their score, displayed as “‘Il tuo punteggio è: ” (“Your score is: ”). In a later stage of the experiment, feedback is withheld, as indicated by the message “Il feedback non-verrà mostrato in questa parte” (“Feedback will not be shown in this section”). Each phase consists of 10 trials, with 20 s allowed for decision-making in each trial, followed by a 10-s period for feedback or feedback omission.

The active task consisted of a numerical version of the Prisoner's Dilemma game (Peterson, 2015), which measured decision-making and social interaction. Participants were divided into pairs and played 20 rounds of the game, during which they had to choose between cooperation and betrayal (see Figure 1 for an outline of the procedure). For the first 10 rounds, participants were given feedback through points gained, thus inferring the decisions of the other participants. For the latter 10 rounds, the game was “blind,” meaning no feedback was provided after each round (Figure 1).

#### 2.3.2 Passive task: standardized presentation

In the passive task, four participants per session listened to a 15-min presentation introducing basic neuroscientific concepts, prepared for a general audience using plain language. The presentation was standardized in terms of timing, features of the presenting agent (i.e., presenter), and material presented across all conditions to ensure consistency; participants were not informed of the topic beforehand. This task assessed participants' attention, retention, and comprehension of the material.

### 2.4 Scenarios

All participants were randomly assigned to one of the four scenarios: 31 to the Presence scenario, 25 to Teams, 25 to the Metaverse VR-, and 21 to the scenario of Metaverse VR+. The count of participants was adjusted to 102 after excluding one individual who identified as non-binary, to ensure that gender analysis was not affected. No significant differences were found in the distribution by age groups (Fisher's Exact Test, *p* = 0.1469) or in terms of gender (χ^2^ = 5.3362, *df* = 3, *p* = 0.15).

#### 2.4.1 Presence

In this workplace scenario, participants interacted in-person. In this study, we define “Presence” as the condition where participants are physically co-located, engaging in face-to-face interactions without any mediated or virtual interfaces For the active task, the four participants were divided into two pairs, with each pair assigned to a separate room. Each participant was assigned to a laptop while facing the other participant. Thus, two participants were placed in one room, and the other two in another room. Each participant was assigned to a laptop while facing their partner, but they were unable to see the other participant's screen. Explicit communication between participants was not allowed, meaning no speech or gestures were permitted during the task. For the passive task, all four participants were in the same room, where a presenting agent was located sharing a presentation. They were asked to passively listen to a presentation.

#### 2.4.2 Teams

In this workplace scenario, participants interacted remotely through a video call, with their cameras on but microphones muted. Each participant was placed in a separate room, simulating a videoconferencing environment. For the active task, the four participants were divided into two pairs, and each pair interacted through a video call. Participants could see each other on the screen but were not allowed to communicate explicitly. No speech or gestures were permitted during the task. For the passive task, all four participants remained in their separate rooms but were connected to the same video call, where the presenter shared the slides for the presentation.

#### 2.4.3 Metaverse without virtual reality (metaverse VR-)

**Figure 2:**
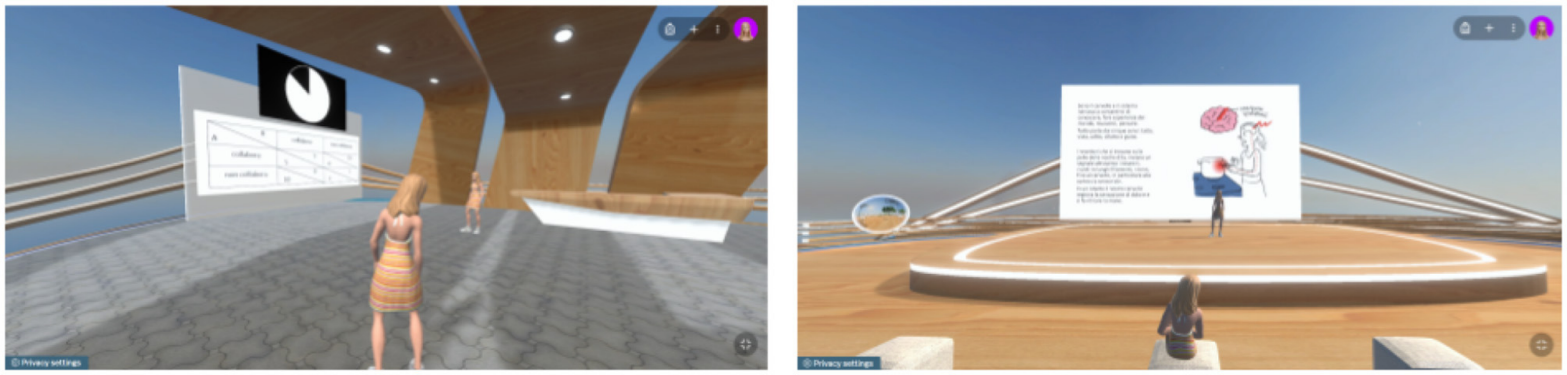
Footage from actual Metaverse VR- condition of active and passive task.

In this workplace scenario, each of the four participants was seated in a distinct room, with two laptops provided to each participant. For the active task, one laptop was used to access the metaverse and control an avatar, while the other laptop displayed the task to be performed. Within the Spatial.io platform, a cutting-edge platform that redefines virtual collaboration by creating immersive 3D spaces where users can meet, work, and interact, two rooms were created, with the four participants divided into two pairs, each pair assigned to a separate metaverse-room to perform the task without interference (Figure 2). In the passive task, the four participants accessed the same virtual room where the presenter's avatar gave the presentation using slides (Figure 2).

#### 2.4.4 Metaverse with virtual reality (metaverse VR+)

**Figure 3:**
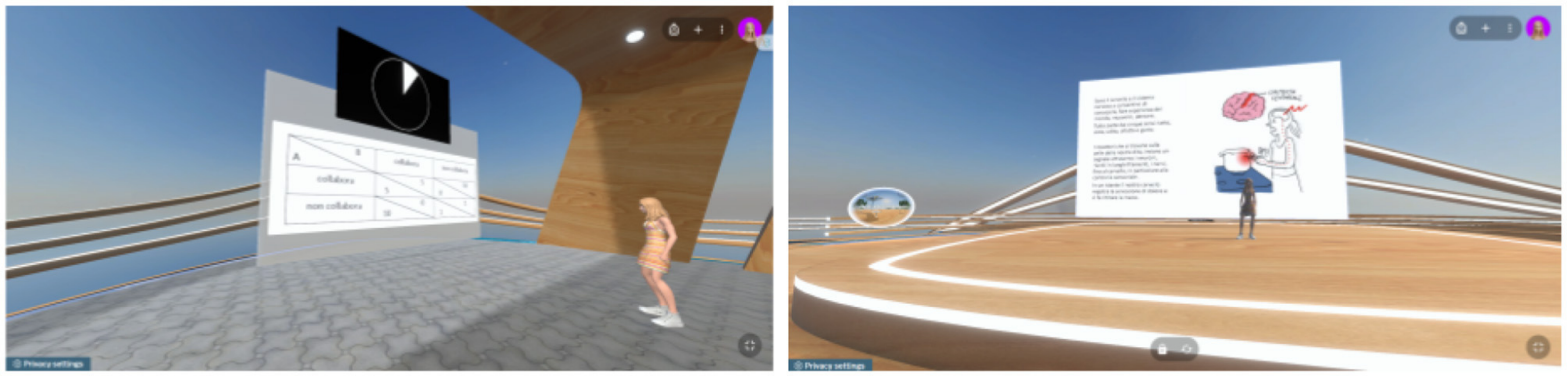
Footage from actual Metaverse VR+ condition of active and passive task.

In this workplace scenario, each of the four participants was seated in a different room and had to use one computer and the all-in-one VR system Oculus Quest 2. To perform the two tasks, participants wore the Oculus Quest 2 and interacted with each other in the virtual environment. For the active task, the four participants were divided into two pairs, each pair placed in a different virtual room as in the previous scenario. While immersed in the virtual world, participants could see each other within the environment, but responses were collected using the laptop keyboard (Figure 3). For the passive task, all four participants accessed the same virtual room, where the presenter's avatar gave the presentation using slides in the Metaverse environment (Figure 3).

### 2.5 Data collection

#### 2.5.1 Behavioral measures

All behavioral data about the task and the post-task assessments were collected using Qualtrics (Version 2023 of Qualtrics.Copyright © 2020).

##### 2.5.1.1 Post-task ergonomic assessment

After completing each task (active and passive), participants completed a short ergonomic questionnaire designed to assess subjective user experience. The questionnaire included five Likert-scale items, addressing: physical comfort during the task, perceived tiredness/fatigue, ease of using the technology and devices, and overall satisfaction with the setup. Participants also responded to one open-ended question, inviting additional feedback or comments about the ergonomic experience.

##### 2.5.1.2 Psychometric and qualitative assessments

In addition to the tasks mentioned above, the administration of psychometric questionnaires and demographic questions has been carried out.

##### 2.5.1.3 Balanced emotional empathy scale

The BEES (Mehrabian, 1996) is a 30-item scale that measures affective empathy-specifically, individuals' emotional responsiveness to the feelings of others. Items are rated on a 9-point Likert scale from -4 (“strongly disagree”) to +4 (“strongly agree”), with higher total scores reflecting higher emotional empathy. Example items include: “Unhappy movie endings haunt me for hours afterward.” I cannot continue to feel okay if others around me are depressed.” The Italian version of the BEES was used in its validated form, without further modification (Meneghini et al., 2012).

##### 2.5.1.4 Ten item personality inventory

To assess personality traits, we used the 10-Item Personality Inventory (Gosling et al., 2003), a validated short-form instrument that captures the Big Five personality dimensions: Openness to Experience, Conscientiousness, Extraversion, Agreeableness, and Emotional Stability. The TIPI consists of 10 items, with two items per trait, rated on a 7-point Likert scale ranging from 1 (“Disagree strongly”) to 7 (“Agree strongly”). Each trait is measured through one positively keyed and one reverse-keyed item. Example items include: “I see myself as extroverted, enthusiastic,” or “I see myself as dependable, self-disciplined.” The Italian version of the TIPI was used in its validated form, without further modification (Chiorri et al., 2014).

#### 2.5.2 Data acquisition and processing of physiological measures

EEG data were recorded using the MUSE 2 headband, which sampled at 256 Hz. The Muse system features electrodes placed at locations corresponding to AF7, AF8 (frontally), TP9, TP10 (temporally), with Fpz serving as the reference electrode. The EEG data were streamed to a connected PC via Bluetooth using the Muse SDK, where they were stored for offline analysis. Post-processing of the EEG data was performed in MATLAB R2023b, using EEGLAB (Delorme and Makeig, 2004) along with custom scripts. The continuous EEG signals were filtered with a dual-pass Butterworth filter (0.1 to 30 Hz) to remove noise and a 60 Hz notch filter to eliminate power line interference. Power spectral density for Theta (*P*_θ_), Alpha (*P*_α_), and Beta (*P*_β_) frequency bands, defined as 4–8 Hz, 8–12 Hz, and 12–30 Hz, respectively, was computed using the pwelch method (Parameshwaran and Thiagarajan, 2019).

Several cognitive and relaxation indices were calculated based on these frequency bands:

**Fatigue index (F)**: an indicator of mental fatigue, computed as:


$$F=\frac{P_{\theta }+P_{\alpha }}{P_{\beta }}$$

This index reflects the balance between lower-frequency brainwaves (associated with relaxation and reduced alertness) and higher-frequency Beta waves (linked to focus and activity) (Eoh et al., 2005).

**Concentration index (C)**: a measure of cognitive focus, derived as:


$$C=\frac{P_{\beta }}{P_{\theta }}$$

This index quantifies the ratio of Beta to Theta power, indicating cognitive effort and attention levels (Doren et al., 2017).

**Relaxation index (R)**: an indicator of relaxation, calculated as:


$$R=\frac{P_{\theta }}{P_{\alpha }}$$

A higher value suggests greater relaxation, as it measures the relationship between Theta (associated with relaxation) and Alpha (linked to calm wakefulness) activity (Nagendra et al., 2015).

These indices were calculated on frontal and temporal electrodes. The selection of frontal and temporal regions is crucial because these areas of the brain are involved in perceptual processing, executive functions, and attention regulation. Specifically, the frontal cortex (monitored via AF7 and AF8 electrodes) is associated with cognitive control, decision-making, and attention (Moslemi and Chalabianloo, 2024), while the temporal regions (monitored via TP9 and TP10 electrodes) are important for memory, emotional regulation, and sensory integration (Jo et al., 2024). The EEG indices (Concentration, Fatigue, and Relaxation) were computed based on spectral power values without normalization. Therefore, index values reflect relative comparisons within the participant sample.

#### 2.5.3 Task-specific cognitive and emotional measures

##### 2.5.3.1 Active task

Cognitive and emotional states during the active task were assessed using multiple measures. Decision-making patterns were assessed through a combination of behavioral, physiological, and self-report measures. Decision-making patterns were analyzed based on cooperation vs. betrayal choices. EEG data collected during the task were used to compute the Concentration Index (C), reflecting cognitive focus and attentional engagement, and the Fatigue Index (F), indicating mental fatigue based on spectral EEG characteristics. Emotional responses were evaluated through post-task questionnaires addressing participants' emotional experiences (e.g., frustration, engagement) and decision-making strategies. Additionally, participants completed the Balanced Emotional Empathy Scale (BEES) (Mehrabian, 1996), a validated measure of affective empathy, to capture individual differences in emotional responsiveness. In addition, participants answer open-ended questions about their decision-making strategy and emotional responses during the game (“We ask you to share your reflections and feelings about the experience.; How did the context influence your perception of the situation and your decisions?; What emotions did you feel during the task?; Is there anything specific about the context that you felt was particularly significant or had an impact on your decisions?; Provide any observations you think might be helpful.”).

##### 2.5.3.2 Passive task

Cognitive engagement was measured through accuracy in answering to 10 multiple-choice questions and summarizing the presentation. Emotional engagement was assessed using self-reported ratings on how interesting, engaging, or boring the presentation felt. EEG data were also collected during the task to assess mental focus using the Concentration Index (C) and relaxation levels with the Relaxation Index (R). These indices provided insight into participants' cognitive effort and emotional responses while listening to the presentation. After listening to the presentation, participants were asked to summarize the content and answer 10 multiple-choice questions that covered key concepts from the presentation, including brain anatomy, neuronal communication, sensory integration, and well-known neuroscience case studies, to check for their comprehension and information retention. The full list of questions is available in the Supplementary material S2.

### 2.6 Statistical analysis

Data analysis was conducted using JASP Version 0.19.1, a graphical statistical software (JASP Team, 2024). A two-way analysis of covariance (ANCOVA) was conducted to test for mean differences among experimental workplace scenarios and between genders on task performance, electrophysiological indices, and self-reported measures, after controlling for age. Separate analyses have been conducted for the active and passive tasks. *Post-hoc* contrasts were performed to examine the effects of gender, age, and scenario on the outcomes. Age and its interactions were tested using linear regression. This allowed for a comprehensive comparison across groups and scenarios, enabling a deeper understanding of the influence of these variables on behavior and physiological responses. Statistical significance was set to *p* < 0.05. Given the naturalistic, workplace-based nature of the experiment, confounding variables (e.g., participants' job position, prior experiences, the time of day, etc.) were not controlled to preserve the ecological validity of the study.

## 3 Results

### 3.1 Active task

**Figure 4:**
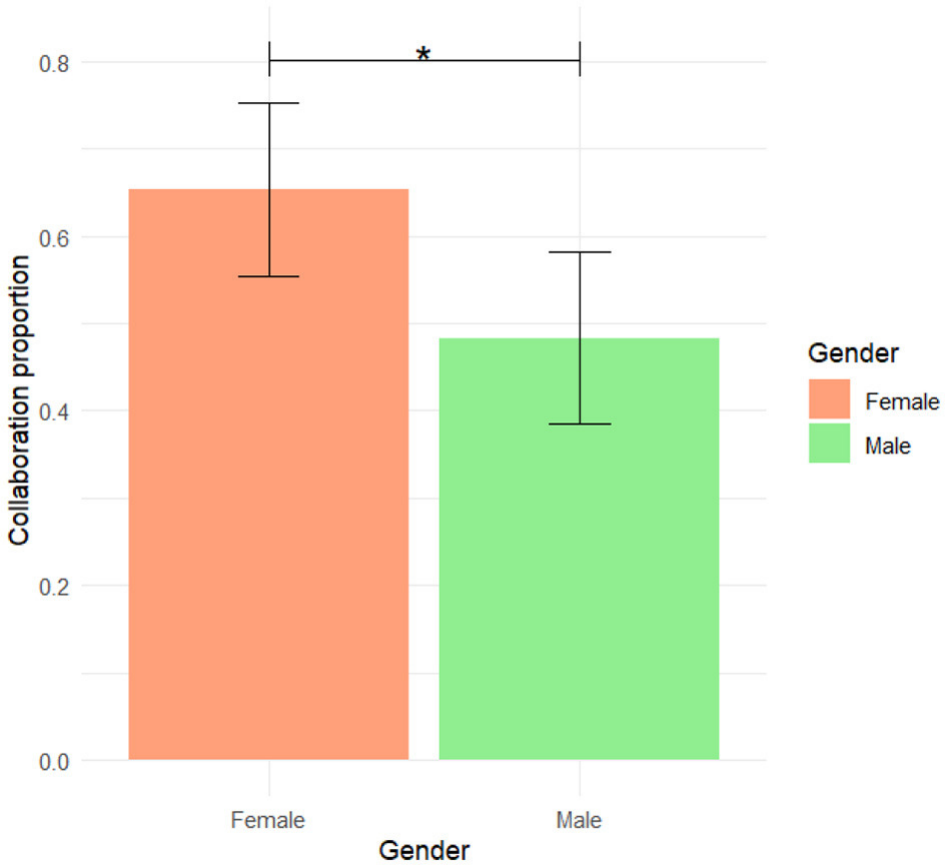
Collaboration proportion by gender. *Indicates statistical significance at *p* < 0.05.

In the active task, no significant differences in the proportion of collaborative choices were observed across workplace scenarios (*F*(3, 60) = 0.278;*p* = 0.841). This suggests that the type of workplace environment, whether physical or virtual, did not directly influence collaborative behavior among participants. However, a significant main effect of gender was found (*F*(1, 60) = 6.559;*p* = 0.013), with female participants demonstrating a higher propensity to collaborate than their male counterparts (Figure 4). Although the interaction between workplace and gender was not statistically significant, an exploratory analysis suggested that this gender difference in collaboration was more pronounced in the Presence workplace than in the virtual ones (Table 2). Age did not have a significant impact on collaborative behavior (*F*(1, 60) = 0.079, *p* = 0.779) (Table 2).

**Table 2:** ANCOVA—collaboration proportion.

**Effect**	**Sum of squares**	**df**	**Mean square**	**F**	**p**	**η^2^**	**ω^2^**
gender	0.539	1	0.539	6.559	0.013	0.092	0.077
workplace	0.068	3	0.023	0.278	0.841	0.012	0.000
age	0.007	1	0.007	0.079	0.779	0.001	0.000
gender * workplace	0.062	3	0.021	0.250	0.861	0.011	0.000
workplace * age	0.241	3	0.080	0.980	0.408	0.041	0.000
Residuals	4.926	60	0.082				

In terms of cognitive engagement, as measured by EEG-derived indexes (see Section 2.5.2 for details on the physiological index), gender exhibited a significant main effect of the concentration index on frontal areas (*F*(1, 63) = 7.530;*p* = 0.008). Female participants showed higher concentration levels than males across all workplace scenarios, suggesting a consistent trend in gender differences related to focus during collaborative tasks. Despite this, the main effect of the workplace scenario on frontal concentration was not significant (*F*(3, 63) = 1.547, *p* = 0.211), indicating that the environment itself did not significantly affect concentration levels. Additionally, the interaction between gender and scenario was also not significant (*F*(3, 63) = 0.248, *p* = 0.862, Table 3).

**Table 3:** ANCOVA—fatigue, concentration and stress/relaxation.

**Index**	**Region**	**Effect**	**F**	**p**	**η^2^**
Fatigue	Frontal	gender	0.006	0.937	9.25 × 10^−5^
		workplace	0.441	0.724	0.020
		age	0.673	0.415	0.010
		gender * workplace	0.545	0.653	0.024
		workplace	0.684	0.565	0.028
		age	0.011	0.917	1.497 × 10^−4^
		gender * workplace	0.932	0.430	0.038
Concentration	Frontal	gender	7.530	0.008	0.097
		workplace	1.547	0.211	0.059
		age	1.095	0.299	0.014
		gender * workplace	0.248	0.862	0.010
	Temporal	gender	0.520	0.474	0.006
		workplace	0.938	0.428	0.031
		age	0.680	0.413	0.008
		gender * workplace	3.129	0.032	0.105
Relaxation	Frontal	gender	2.751	0.102	0.038
		workplace	0.550	0.650	0.023
		age	0.018	0.894	2.48 × 10^−4^
		gender * workplace	1.174	0.327	0.049
		workplace	0.447	0.720	0.017
		age	0.145	0.705	0.002
		gender * workplace	2.339	0.082	0.091

Interestingly, for the concentration index on temporal regions, a significant interaction between gender and workplace was observed (*F*(3, 63) = 3.129;*p* = 0.032). This suggests that the effect of the scenario on concentration varied depending on gender, with some gender-specific differences emerging depending on the scenario. *Post-hoc* analyses were conducted to further investigate this interaction; however, none of the pairwise comparisons reached statistical significance after adjusting for multiple comparisons. This implies that while the interaction was statistically significant, the differences between specific gender and scenario combinations were not robust when corrected for multiple testing. Neither significant effects nor interactions of the workplace and gender were found on frontal or temporal fatigue and relaxation indices.

Additionally, participant ratings on the ergonomics questionnaire, particularly regarding the ease of use of the devices, had a significant effect on frontal concentration (*F*(1, 63) = 10.074;*p* = 0.002). This finding highlights that ease of use positively influenced concentration during the task, suggesting that interface usability plays a crucial role in maintaining participant focus.

Lastly, two additional ANCOVAs were conducted to assess the role of personality traits and ergonomic factors in influencing the propensity to collaborate (Table 4). The first model, which included personality traits—including Openness, Conscientiousness, Extraversion, Agreeableness, and Emotional Stability—as covariates, found no significant effect (*p*>0.200). This indicates that individual personality differences did not substantially impact participants' willingness to collaborate. Similarly, the second model, which examined ergonomic factors—comfort, tiredness, ease of use, and satisfaction—also showed no significant effects in collaboration (*p*>0.100), suggesting that ergonomic perceptions did not meaningfully influence participants' collaborative behavior during the active task.

**Table 4:** ANCOVA—collaboration proportion, personality traits and ergonomic factors.

**Effect**	**Results**					
	**Sum of squares**	**df**	**Mean square**	**F**	**p**	**η^2^**
Workplace	0.183	3	0.061	0.690	0.562	0.030
Openness	7.970 × 10^−4^	1	7.970 × 10^−4^	0.009	0.925	1.327 × 10^−4^
Conscientiousness	0.064	1	0.064	0.719	0.400	0.011
Extraversion	0.030	1	0.030	0.341	0.561	0.005
Agreeableness	0.034	1	0.034	0.388	0.536	0.006
Emotional stability	0.128	1	0.128	1.452	0.233	0.021
Residuals	5.567	63	0.088			
Workplace	0.059	3	0.020	0.237	0.870	0.010
Comfort	0.194	1	0.194	2.345	0.131	0.032
Tiredness	0.050	1	0.050	0.608	0.439	0.008
Easy of use	0.091	1	0.091	1.094	0.300	0.015
Satisfaction	0.123	1	0.123	1.483	0.228	0.020
Workplace * Satisfaction	0.487	3	0.162	1.960	0.129	0.080
Residuals	5.048	61	0.083			

### 3.2 Passive task

On average, participants answered 8.34 out of 10 comprehension questions correctly (SD = 1.29), indicating a generally high level of content retention. Performance was relatively consistent across workplace scenarios: participants in the Presence condition had the highest average score (*M* = 8.57, SD = 1.14), followed by Teams (*M* = 8.36, SD = 0.90), Metaverse no VR (*M* = 8.25, SD = 1.42), and Metaverse VR (*M* = 8.05, SD = 1.72). An ANCOVA was conducted to examine the effects of different workplaces – Presence, Teams, Metaverse VR-, and Metaverse VR+ – on performance on the passive task. The analysis revealed that the main effect of the workplace on performance was not statistically significant, (*F*(3, 70) = 0.433;*p* = 0.730), suggesting that the workplace alone did not directly influence the accuracy of participants' responses (see Table 5). However, a significant interaction between workplace and age was observed (*F*(3, 70) = 4.104;*p* = 0.01). This suggests that age moderates the effect of the workplace on performance, indicating that participants of different ages performed differently across workplace scenarios.

**Table 5:** ANCOVA—correct responses.

**Cases**	**Sum of squares**	**df**	**Mean square**	**F**	**p**	**η^2^**	**ω^2^**
workplace	0.021	3	0.007	0.433	0.730	0.015	0.000
age	0.036	1	0.036	2.270	0.136	0.025	0.014
gender	0.012	1	0.012	0.757	0.387	0.008	0.000
workplace * gender	0.046	3	0.015	0.955	0.419	0.032	0.000
workplace * age	0.196	3	0.065	4.104	0.010	0.138	0.103
Residuals	1.117	70	0.016				

**Figure 5:**
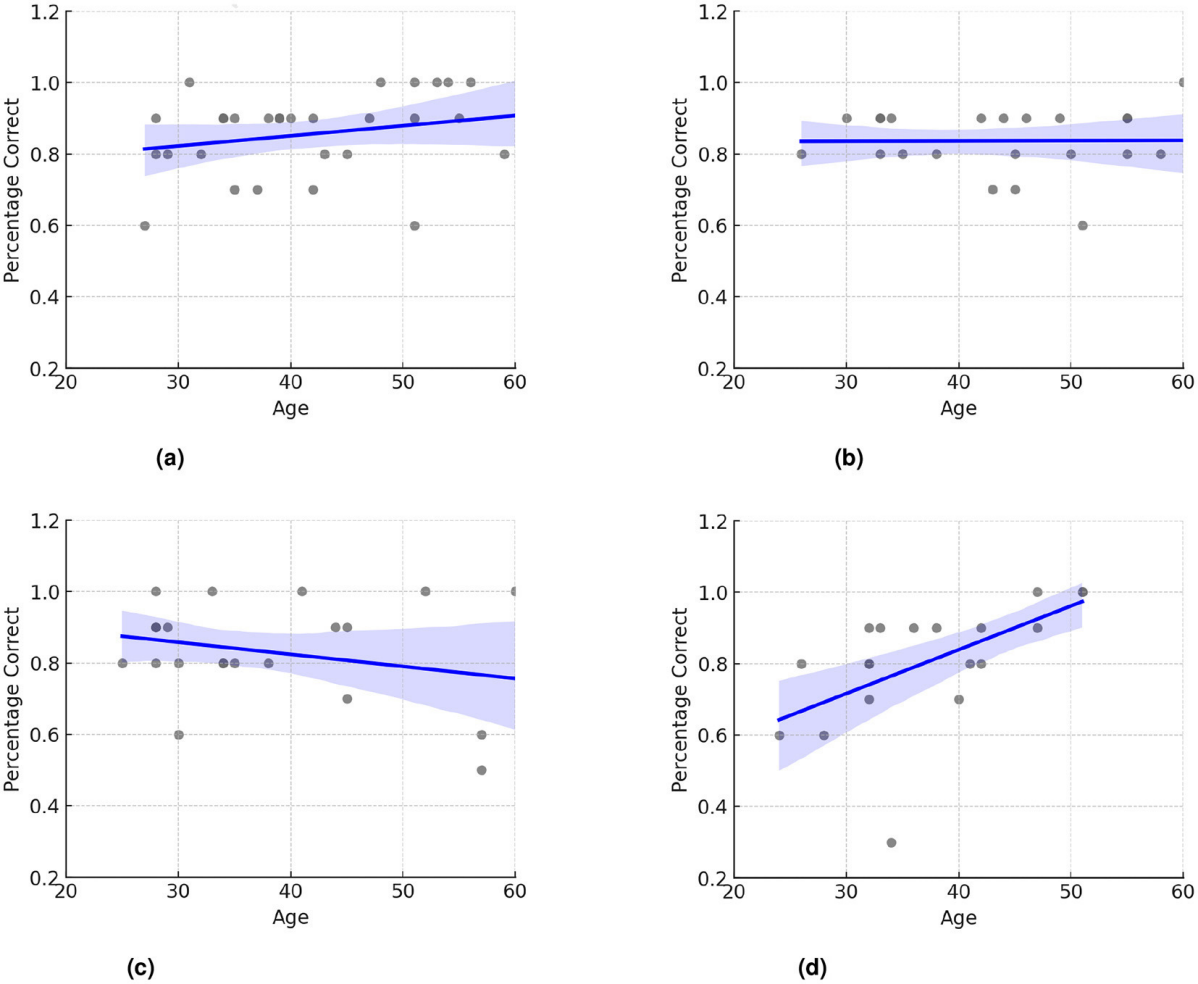
Descriptive graphs for age in each of the 4 workplaces. **(a)** Presence workplace. **(b)** Teams workplace. **(c)** Metaverse VR- workplace. **(d)** Metaverse VR workplace.

A *post-hoc* linear regression analysis was conducted to investigate this interaction further. This analysis showed a significant interaction effect between age and workplace in the Metaverse VR+ (β = 0.013;*p* = 0.001), indicating that older participants performed better in this scenario across workplace scenarios (Figure 5).

Regarding EEG-derived cognitive engagement (see Section 2.5.2 for details on the physiological index), no significant effects of the workplace were found on frontal fatigue (*F*(3, 69) = 0.292, *p* = 0.831). However, self-reported tiredness was significantly associated with frontal fatigue levels (*F*(1, 69) = 4.171;*p* = 0.045), indicating that subjective reports of tiredness were reliable indicators of cognitive strain during the task. In contrast, temporal fatigue revealed a significant main effect of age (*F*(1, 69) = 4.782;*p* = 0.032), with older participants showing greater fatigue. However, no significant effects of the workplace on temporal fatigue were found (*F*(3, 69) = 0.116;*p* = 0.950), suggesting that age, rather than the environment, had a stronger influence on fatigue during the passive task (see Table 6).

**Table 6:** ANCOVA—fatigue, concentration and relaxation.

**Index**	**Region**	**Effect**	**Sum of squares**	**F**	**p**	**η^2^**
Fatigue	Frontal	workplace	3.570	0.292	0.831	0.020
		gender	7.662	1.880	0.175	0.027
		age	1.028	0.252	0.617	0.004
		gender * workplace	4.640	0.379	0.768	0.016
		gender	2.387	0.411	0.524	0.013
		age	27.764	4.782	0.032	0.064
		gender * workplace	13.121	0.753	0.524	0.032
Concentration	Frontal	workplace	13.430	5.570	0.002	0.173
		gender	6.728	8.371	0.005	0.087
		age	0.352	0.437	0.511	0.005
		gender * workplace	0.661	0.274	0.844	0.009
		gender	0.283	0.853	0.359	0.011
		age	0.194	0.585	0.447	0.007
		gender * workplace	0.607	0.609	0.611	0.023
Relaxation	Frontal	workplace	4.196	0.643	0.590	0.026
		gender	0.006	0.003	0.959	3.62 × 10^−5^
		age	0.359	0.165	0.686	0.002
		gender * workplace	2.608	0.400	0.754	0.016
		gender	0.072	0.053	0.819	5.92 × 10^−4^
		age	3.294	2.409	0.125	0.027
		gender * workplace	5.711	1.392	0.253	0.047

Interestingly, frontal concentration levels showed a significant main effect of workplace (*F*(3, 69) = 5.570;*p* = 0.002). *Post-hoc* comparisons indicated that participants in the Metaverse VR+ condition exhibited significantly lower frontal concentration levels compared to those in Presence (*p* = 0.004) and Metaverse VR- (*p* = 0.003) conditions. This suggest that the fully immersive VR environment may have negatively impacted participants' ability to concentrate during the passive task. Gender also had a significant effect on frontal concentration (*F*(1, 69) = 8.371;*p* = 0.005), with female participants showing higher levels of frontal concentration than males across all workplaces. However, no significant interaction between workplace and gender was observed (*F*(3, 69) = 0.274;*p* = 0.844), indicating that gender differences in concentration were consistent across environments (see Table 6).

In terms of temporal concentration, the effect of the workplace was not statistically significant (*F*(3, 69) = 2.210;*p* = 0.095), though the results tended toward a potential difference with lower concentration levels in Metaverse VR+ condition.

Regarding stress levels, no significant main effects of the workplace scenario were observed for frontal stress (*F*(3, 69) = 0.643;*p* = 0.590), nor gender (*F*(1, 69) = 0.003;*p* = 0.959) or age (*F*(1, 69) = 0.165;*p* = 0.686). Similarly, no significant differences in temporal stress were detected between workplace scenarios (*F*(3, 69) = 2.089;*p* = 0.110). However, satisfaction, as measured by responses to the ergonomic questionnaire, had a significant effect on temporal relaxation (*F*(1, 69) = 4.404;*p* = 0.040). This suggests that higher satisfaction with the ergonomic setup was associated with greater relaxation and lower stress levels, highlighting the importance of ergonomic factors in virtual environments (see Table 6).

Finally, an additional ANCOVA was performed to assess the role of ergonomic factors-including comfort, tiredness, ease of use, and satisfaction—on task performance. None of these factors reached statistical significance (*p*>0.300), suggesting that ergonomic perceptions did not significantly influence participants' performance in the passive task (Table 7).

**Table 7:** ANCOVA—correct responses and ergonomic factors.

**Cases**	**Sum of squares**	**df**	**Mean square**	**F**	**p**	**η^2^**
Satisfaction	0.014	1	0.014	0.770	0.383	0.010
Workplace	0.019	3	0.006	0.340	0.797	0.013
Comfort	0.011	1	0.011	0.572	0.452	0.007
Tiredness	1.587 × 10^−5^	1	1.587 × 10^−5^	8.520 × 10^−4^	0.977	1.115 × 10^−5^
Easy of use	0.001	1	0.001	0.059	0.808	7.747 × 10^−4^
Residuals	1.378	74	0.019			

## 4 Discussion

This study investigated how different workplace environments—physical presence, videoconferencing (Teams), non-immersive Metaverse (Metaverse VR-), and immersive Metaverse (Metaverse VR+)—may impact task performance, cognitive engagement, comfort, and social dynamics during collaborative and passive tasks. By examining both behavioral and electrophysiological brain responses across these environments, this study assessed whether virtual platforms can not only serve as effective alternatives to traditional workplaces but also act as transformative tools for enhancing collaboration and engagement. The results suggest that while virtual environments can replicate several aspects of physical presence, there are key differences in how participants respond to these settings. These observations provide valuable insights into the potential advantages and challenges of using virtual platforms like the Metaverse in professional settings, with implications for future workplace design and remote collaboration.

The physical presence environment is traditionally viewed as the ideal setting for collaboration due to its ability to facilitate direct interaction. However, in a post-pandemic world, where flexible and remote work has become the norm, virtual environments are a valid alternative (Dyba and Di Maria, 2024; Kähkönen, 2023). Our findings suggest that virtual platforms such as Microsoft Teams and the Metaverse can effectively replicate the interactive experience of physical presence, enabling team members to collaborate efficiently or to promote learning sessions, even when spatially dispersed. These findings support the growing trend toward remote work and highlight the potential of virtual environments to foster collaboration without the need for physical proximity (Bayro et al., 2022; Hoppe et al., 2018).

One of the key findings of this study was the role of gender in shaping both collaboration and cognitive engagement. Across all workplace scenarios, female participants exhibited higher levels of concentration, as measured by EEG, and a greater propensity to collaborate than their male counterparts, particularly in the physical presence environment. These findings are consistent with previous research suggesting that women engage more deeply in social and collaborative tasks, potentially due to greater attentional focus and social interaction skills (Emad et al., 2017; Peshkovskaya et al., 2018). Importantly, this gender-based effect on the EEG-based index of frontal concentration was statistically significant across all workplace settings, suggesting that it is a robust pattern rather than a context-specific anomaly. While gender differences in cognitive engagement are well-documented (Andreano and Cahill, 2009; Guiso et al., 2008; Weiss et al., 2003), additional factors may have influenced this outcome in our study, such as familiarity with the digital tools, motivational differences, or variations in cognitive strategy. Future research should explore these potential confounders in more detail, possibly through mixed-method approaches including interviews or longitudinal tracking of task engagement. In addition, the significant interaction between gender and workplace on the EEG-based index of temporal concentration during the active task—though not reflected in *post-hoc* comparisons—points to possible subtle differences in how males and females allocate attentional resources depending on the environment. Temporal EEG activity is also associated with processing social cues and language, both of which may be perceived differently in immersive vs. non-immersive contexts (Gregory et al., 2021; Soni et al., 2024; Tremmel et al., 2019). The lack of robust follow-up effects could be due to sample size limitations or insufficient sensitivity of these EEG indices. These findings of our field experiment suggest the need for studies with larger and more balanced samples, or repeated-measures designs, to clarify how gender and environment interact in shaping attention and cognitive load. Encouraging the use of virtual platforms may foster more active engagement and equitable collaboration across diverse teams. The consistently higher concentration levels observed among female participants suggest that virtual environments can support a higher engagement, particularly for users who may be more attuned to social or collaborative dynamics. However, these effects likely interact with other factors beyond gender alone (Darics and Gatti, 2019). The lack of significant interaction between workplace and gender suggests that virtual platforms can help promote equitable collaboration across teams, regardless of gender, age, or other demographic variables (Wu and Kane, 2016).

Contrary to the common belief that virtual reality environments induce greater fatigue (Souchet et al., 2023; Wang et al., 2020), our results showed no significant increase in perceived fatigue across the different settings. However, EEG-derived measures of brain activity revealed that the immersive nature of the Metaverse VR+, particularly during the passive task, was associated with lower concentration levels compared to both the physical presence and Metaverse VR- scenarios. This could be due to the novelty of immersive technologies like VR, which, especially for less experienced users, could contribute to reduce concentration, as participants might be more focused on exploring the virtual environment rather than performing the task (Soroko and Lytvynova, 2021). Additionally, while the interaction between gender and workplace on temporal concentration during the active task did not yield significant *post-hoc* differences, the significant interaction term itself suggests that subtle variations may exist across workplace scenarios. Temporal brain regions are associated with auditory and social information processing (Deen et al., 2015; Meyer and Lieberman, 2018; Olson et al., 2013), and it is plausible that immersive vs. non-immersive environments activate these areas differently for men and women. The absence of significant pairwise comparisons could reflect a lack of statistical power or limited measurement sensitivity. Future research could benefit from larger sample sizes, within-subject designs, or finer-grained EEG measures to better capture interaction effects and underlying neurocognitive dynamics. Also, when we talk about virtual reality and immersive environments, ergonomic issues, such as cybersickness, make it harder for users to complete the task (Klippel, 2020). Moreover, even though immersive technologies can help focus attention by isolating specific objects or concepts while reducing external distractions, the study of Soroko and Lytvynova (2021) showed that this benefit is more pronounced when users are familiar with the technology.

Age also played a critical role in moderating performance across different environments. Older participants performed better than their younger counterparts in the passive task, particularly in the Metaverse VR+ condition. This finding challenges the assumption that younger, more digitally savvy individuals naturally excel in virtual environments (Tomasi et al., 2018). Instead, it appears that older participants, perhaps due to a more solid experience in maintaining focus in less dynamic settings, were better equipped to perform passive tasks like information retention (Mager et al., 2005). Younger participants, on the other hand, may require more interactive and stimulating features to stay engaged, especially in less immersive tasks (Petruse et al., 2024). This age-related trend underscores the importance of tailoring virtual environments to the specific needs of different demographic groups, ensuring both younger and older users can engage effectively depending on the task type and environment. This trend was not observed in the active task, where age did not significantly influence collaboration or task performance. Given the distinct engagement patterns observed across different age groups, virtual environment designers should consider customizable settings that can be adapted to the user's age and task requirements. For instance, integrating more interactive features for younger users while offering focused, less immersive content for older users could optimize both learning and performance outcomes. While older participants seemed more adept at passive tasks in VR environments, younger participants may benefit from more interactive and engaging elements that suit their preferences for dynamic digital interactions. Therefore, designing virtual spaces that account for user age and task complexity is crucial for optimizing performance and engagement across different types of activities.

Although ergonomic ratings did not significantly differ across workplace scenarios, our results revealed a meaningful association between ergonomic satisfaction and EEG-based relaxation indices during the passive task. Participants who reported greater satisfaction with the ergonomic setup exhibited significantly higher relaxation levels in temporal brain regions. This suggests that perceived comfort can influence physiological markers of emotional and cognitive ease. These findings underscore the importance of ergonomic design—particularly in immersive environments where discomfort may be more likely. Future implementations of VR-based workspaces should prioritize lightweight hardware, intuitive interfaces, and personalized adjustments to enhance user comfort and mitigate cognitive strain, even in relatively short sessions.

Our results also provide important insights into cognitive load and fatigue in virtual environments. While participants did not report significantly higher fatigue in the immersive VR scenarios, self-reported tiredness was significantly associated with increased levels of EEG-measured frontal fatigue, suggesting that subjective experiences of tiredness may be reliable indicators of cognitive strain. Interestingly, EEG data indicated increased brain activity, suggesting a heightened cognitive load in these environments. This discrepancy suggests that users may not consciously perceive increased fatigue, even though the immersive nature of VR places greater demands on their cognitive resources. Increased frontal fatigue in the VR scenarios also aligns with this heightened cognitive load, as revealed by EEG data. These findings suggest that virtual platforms, especially immersive ones, should prioritize user-friendly interfaces and ergonomic design to minimize cognitive overload.

In addition, mental effort, especially during tasks that require concentration and attentiveness, could also be associated with the lack of non-verbal cues in virtual environments such as VR, where avatars are involved. Contrary to face-to-face interactions, and even in videoconferencing, avatars cannot easily reveal facial expressions and body language, crucial for smooth social cognition (Frith, 2009; Sung et al., 2011). The absence of these cues may make it harder for users to interpret others' intentions and navigate the task, subtly increasing mental effort. Additionally, virtual spaces often feature rich visual and auditory stimuli, which, while immersive, can risk overwhelming users and diverting attention from the primary task (Grimshaw-Aagaard and Walther-Hansen, 2024). To address these challenges, the design of virtual platforms should prioritize simplicity and intuitive user interfaces to reduce cognitive strain. This is particularly important for tasks requiring sustained attention, such as passive information retention or learning. A minimalist, distraction-free design can help users focus on the task, improving cognitive ergonomics and overall user experience. Additionally, optimizing the sensory environment by limiting unnecessary visual and auditory elements can further reduce the risk of being overstimulating, enabling participants to maintain higher levels of concentration for longer periods (Kaplan-Rakowski et al., 2024; Kim and Lee, 2022).

While this on field study offers important insights into the cognitive and social dynamics of virtual collaboration, some limitations should be considered. The sample was drawn from a single organization, which may limit the generalizability of findings to broader workplace contexts. Additionally, potential source of bias stems from the fact that participants were colleagues within the same company; existing interpersonal familiarity or team dynamics may have influenced collaboration behavior, particularly during the active task. Finally, environmental and contextual variables—such as time of day, workplace setting, or varying levels of participant motivation—were not controlled in order to preserve ecological validity.

Future studies could benefit from including balanced groups—participants with prior virtual reality (VR) experience and those without—to examine how familiarity with immersive environments influences collaborative behavior and cognitive engagement. This distinction may reveal important differences in how individuals adapt to virtual settings, especially in terms of task performance, communication style, and comfort level within immersive interfaces.

Taken together, the findings from both the active and passive tasks underscore the potential of virtual platforms to enhance remote collaboration and learning. While virtual environments can replicate many aspects of physical presence and promote more inclusive team dynamics, their design must carefully balance immersive features with user comfort and cognitive ergonomics. Future iterations of virtual platforms must prioritize not only engagement but also user wellbeing by reducing cognitive strain and enhancing inclusivity. By integrating adaptive features that cater to various demographic needs, designers can create environments that promote long-term productivity, satisfaction, and inclusivity in increasingly digital workplaces. Further research should continue exploring how specific features, such as personalized avatars and interaction modes, affect collaboration and concentration across diverse user groups.

## 5 Conclusions

In conclusion, this study demonstrates the potential of virtual platforms, such as videoconferencing and the metaverse, to serve as effective alternatives to traditional in-person work environments, while also offering transformative possibilities for enhancing collaboration, engagement, and inclusivity. While virtual platforms can replicate many aspects of physical presence, key differences in cognitive engagement, comfort, and performance emerged, influenced by factors such as gender, age, and task type. Notably, immersive environments offer enhanced opportunities for interaction and creativity but may also introduce challenges related to cognitive load, concentration, and ergonomic discomfort. These findings underscore the importance of designing virtual environments that prioritize user comfort, cognitive ergonomics, and adaptability to individual needs. Future developments should focus on creating customizable, user-friendly interfaces that cater to diverse demographic groups, while ensuring a balance between immersion and ease of use. In practical terms, this entails: (1) matching the level of immersion to task type (e.g., using immersive VR for active collaboration but opting for simpler interfaces in passive learning); (2) enhancing interface usability and cognitive ergonomics to support sustained attention; (3) leveraging virtual platforms to reduce disparities in participation (e.g., gender-based differences observed in physical settings); and (4) ensuring technical reliability and accessibility, including stable connectivity and user-ready equipment. By addressing these factors, virtual platforms have the potential to not only support effective remote collaboration but also to shape the future of work in a rapidly digitalizing world. Further research is essential to refine these tools and fully understand their long-term implications for productivity, wellbeing, and workplace dynamics.

## Data Availability

The datasets presented in this article are not readily available because the dataset used and analyzed in this study was collected as part of the research; however, the data is not owned by the authors and is subject to restrictions. The authors do not have the right to share or distribute the dataset. Requests to access the datasets should be directed to irene.sanchez@imtlucca.it.

## References

[B1] Ali M. Naeem F. Kaddoum G. Hossain E. (2023). Metaverse communications, networking, security, and applications: research issues, state-of-the-art, and future directions. IEEE Commun. Surv. Tutor. 25, 3083–3132. 10.1109/COMST.2023.3347172

[B2] Andreano J. M. Cahill L. (2009). Sex influences on the neurobiology of learning and memory. Learn. Mem. 16, 248–266. 10.1101/lm.918309 19318467

[B3] Bayro A. Ghasemi Y. Jeong H. (2022). “Subjective and objective analyses of collaboration and co-presence in a virtual reality remote environment,” in 2022 IEEE Conference on Virtual Reality and 3D User Interfaces Abstracts and Workshops (VRW) (Christchurch: IEEE), 485–487. 10.1109/VRW55335.2022.00108

[B4] Becchetti L. Salustri F. Solferino N. (2024). The new industrial revolution: the optimal choice for flexible work companies. J. Econ. Interact. Coord. 20, 237–271. 10.1007/s11403-024-00418-y

[B5] Bender Maritan F. Duarte Soliani R. Reis Drumond T. D. Dantas Montilha Satrapa H. F. de Souza Oliveira P. R. Bezerra de Lima Júnior F.. (2024). Cost analysis in the transition of projects to remote work during the COVID-19 pandemic. Environ. Soc. Manag. J./Rev. Gest. Soc. Ambient. 18:e014. 10.24857/rgsa.v18n1-014

[B6] Cali U. Kuzlu M. Karaarslan E. Jovanovic V. (2022). “Opportunities and challenges in metaverse for industry 4.0 and beyond applications,” in 2022 IEEE 1st Global Emerging Technology Blockchain Forum: Blockchain and Beyond, iGETblockchain (New York, NY: IEEE). 10.1109/iGETblockchain56591.2022.10087104

[B7] Cheng J. Bernstein M. Danescu-Niculescu-Mizil C. Leskovec J. (2017). “Anyone can become a troll: causes of trolling behavior in online discussions,” in Proceedings of the 2017 ACM Conference on Computer Supported Cooperative Work and Social Computing (Portland, OR: ACM), 1217–1230. 10.1145/2998181.2998213 PMC5791909 29399664

[B8] Chiorri C. Bracco F. Piccinno T. Modafferi C. Battini V. (2014). Psychometric properties of a revised version of the ten item personality inventory. Eur. J. Psychol. Assess. 31, 109–119. 10.1027/1015-5759/a000215

[B9] Darics E. Gatti M. (2019). Talking a team into being in online workplace collaborations: the discourse of virtual work. Discourse Stud. 21, 237–257. 10.1177/1461445619829240

[B10] Deen B. Koldewyn K. Kanwisher N. Saxe R. (2015). Functional organization of social perception and cognition in the superior temporal sulcus. Cereb. Cortex 25:4374–4387. 10.1093/cercor/bhv111 26048954 PMC4816802

[B11] Delorme A. Makeig S. (2004). EEGLAB: an open source toolbox for analysis of single-trial eeg dynamics including independent component analysis. J. Neurosci. Methods 134, 9–21. 10.1016/j.jneumeth.2003.10.009 15102499

[B12] Doren J. V. Heinrich H. Bezold M. Reuter N. Kranz O. (2017). Theta/beta neurofeedback in children with adhd: feasibility of a short-term setting and plasticity effect. Int. J. Psychophysiol. 112, 80–88. 10.1016/j.ijpsycho.2016.11.004 27829128

[B13] Dyba W. Di Maria E. (2024). Assessment and support of the digitalization of businesses in europe during and after the covid-19 pandemic. Reg. Sci. Policy Pract. 16:12717. 10.1111/rsp3.12717

[B14] Emad M. Neumann D. L. Abel L. (2017). Attentional focus strategies used by regular exercisers and their relationship with perceived exertion, enjoyment, and satisfaction. J. Hum. Sport Exerc. 12, 106–118. 10.14198/jhse.2017.121.09

[B15] Eoh H. J. Chung M. K. Kim S.-H. (2005). Electroencephalographic study of drowsiness in simulated driving with sleep deprivation. Int. J. Ind. Ergon. 35, 307–320. 10.1016/j.ergon.2004.09.006

[B16] Fortuna F. Rossi L. Elmo G. C. Arcese G. (2023). Italians and smart working: a technical study on the effects of smart working on the society. Technol. Forecast. Soc. Change 187:122220. 10.1016/j.techfore.2022.122220

[B17] Frith C. (2009). Role of facial expressions in social interactions. Philos. Trans. R. Soc. B Biol. Sci. 364, 3453–3458. 10.1098/rstb.2009.0142 19884140 PMC2781887

[B18] Gosling S. D. Rentfrow P. J. Swann Jr W. B. (2003). A very brief measure of the big five personality domains. J. Res. Pers. 37, 504–528. 10.1016/S0092-6566(03)00046-1

[B19] Gregory S. Wang H. Kessler K. (2021). Eeg alpha and theta signatures of socially and non-socially cued working memory in virtual reality. Psychophysiology 58:e13988. 10.31234/osf.io/mkags PMC9164206 34894148

[B20] Grimshaw-Aagaard M. Walther-Hansen M. (2024). Less-is-more: auditory strategies for reduced reality. Pers. Ubiquitous Comput. 28, 713–725. 10.1007/s00779-024-01808-6

[B21] Guiso L. Monte F. Sapienza P. Zingales L. (2008). Culture, gender, and math. Science 320, 1164–1165. 10.1126/science.1154094 18511674

[B22] Harthy B. A. Harthi A. A. Arianpoor A. Zaidan A. S. (2023). “Impact of metaverse at workplace: opportunity and challenges,” in International Multi-Disciplinary Conference-Integrated Sciences and Technologies (Cham. Springer Nature Switzerland), 54–68. 10.1007/978-3-031-51300-8_4

[B23] Higuchi K. Chen Y. Chou P. A. Zhang Z. Liu Z. (2015). “Immerseboard: Immersive telepresence experience using a digital whiteboard,” in Proceedings of the 33rd Annual ACM Conference on Human Factors in Computing Systems (Seoul: ACM), 2383–2392. 10.1145/2702123.2702160

[B24] Hoppe A. H. Reeb R. van de Camp F. Stiefelhagen R. (2018). “Interaction of distant and local users in a collaborative virtual environment,” in Virtual, Augmented and Mixed Reality: Interaction, Navigation, Visualization, Embodiment, and Simulation: 10th International Conference, VAMR 2018, Held as Part of HCI International 2018, Las Vegas, NV, USA, July 15-20, 2018, Proceedings, Part I (Springer International Publishing: New York), 328–337. 10.1007/978-3-319-91581-4_24

[B25] JASP Team (2024). JASP (Version 0.19.0) [*Computer software*]. Available online at: https://jasp-stats.org/

[B26] Jo H-J. Park C. Lee E. Lee J. Kim J. Han S.. (2024). Neural effects of one's own voice on self-talk for emotion regulation. Brain Sci. 14:637. 10.3390/brainsci14070637 39061378 PMC11274574

[B27] Kähkönen T. (2023). Remote work during the COVID-19 pandemic: identification of working life impacts, employees' data protection abilities and trust outcomes. J. Organ. Change Manag. 36, 472–492. 10.1108/JOCM-06-2022-0179

[B28] Kalra D. Kota S. Alomari G. I. Afifi M. A. Mushtaha A. S. (2023). “Toward smart organization: the metaverse as breakthrough for learning organization,” in 2023 International Conference on Business Analytics for Technology and Security (ICBATS) (Dubai: IEEE), 1–5. 10.1109/ICBATS57792.2023.10111246

[B29] Kaplan-Rakowski R. Cockerham D. Ferdig R. E. (2024). The impact of sound and immersive experience on learners when using virtual reality and tablet: a mixed-method study. Br. J. Educ. Technol. 55, 1560–1582. 10.1111/bjet.13417

[B30] Karim E. Pavel H. R. Nikanfar S. Hebri A. Roy A. Nambiappan H. R.. (2024). Examining the landscape of cognitive fatigue detection: a comprehensive survey. Technologies 12:38. 10.3390/technologies12030038

[B31] Kim H. Lee I.-K. (2022). Studying the effects of congruence of auditory and visual stimuli on virtual reality experiences. IEEE Trans. Vis. Comput. Graph. 28, 2080–2090. 10.1109/TVCG.2022.3150514 35167477

[B32] Klippel A. (2020). From spatial to platial—the role and future of immersive technologies in the spatial sciences. J. Spat. Inf. Sci. 21, 33–45. 10.5311/JOSIS.2020.21.722

[B33] Kritika. (2024). “Embarking on the digital frontiers of metaverse,” *Exploring the Use of Metaverse in Business and Education* (Hershey, PA: IGI Global). 10.4018/979-8-3693-5868-9.ch002

[B34] Lee J. (2023). “The impact of metaverse technology use on team creativity in virtual teams,” in Proceedings of the 29th Americas Conference on Information Systems (AMCIS 2023) (Atlanta, GA: Association for Information Systems).

[B35] Mager R. Falkenstein M. Störmer R. Brand S. Müller-Spahn F. Bullinger A. (2005). Auditory distraction in young and middle-aged adults: a behavioural and event-related potential study. J. Neural Transm. 112, 1165–1176. 10.1007/s00702-004-0258-0 15614427

[B36] Mandala V. Reetha Jeyarani M. Kousalya A. Arumugam M. (2023). “An innovative development with multidisciplinary perspective in metaverse integrating with blockchain technology with cloud computing techniques,” in Proceedings of the 6th International Conference on Inventive Computation Technologies, ICICT 2023—*Proceedings* (New York, NY: IEEE). 10.1109/ICICT57646.2023.10134108

[B37] Massari F. Van Belle J. P. Turpin M. (2024). “Navigating new realities: experiences of early adopters in the metaverse,” in Proceedings of the 2024 International Conference on Advanced Visual Interfaces, 1–3. 10.1145/3656650.3656702

[B38] Matar S. Shaker A. Mahmud S. Kim J. H. van't Klooster J. W. (2022). “Design of a mixed reality-based immersive virtual environment system for social interaction and behavioral studies,” in International Conference on Intelligent Human Computer Interaction (Cham: Springer Nature Switzerland), 201–212. 10.1007/978-3-031-27199-1_21

[B39] Mehrabian A. (1996). Manual for the Balanced Emotional Empathy Scale (BEES). Available from Albert Mehrabian. 1130. Monterey, CA.

[B40] Meneghini A. M. Sartori R. Cunegatti L. (2012). Adattamento Italiano Della Balanced Emotional Empathy Scale (BEES) di Albert Mehrabian. Unpublished manuscript, Università di Padova.

[B41] Meyer M. L. Lieberman M. D. (2018). Social working memory: neurocognitive networks and directions for future research. Front. Psychol. 9:2395. 10.3389/fpsyg.2012.0057 23267340 PMC3527735

[B42] Moslemi B. Chalabianloo G. (2024). The effectiveness of transcranial direct current stimulation over prefrontal cortex on attention, working memory, decision-making, social cognition and quality of life in older adults. Aging Psychol. 9, 399–417. 10.22126/jap.2024.9693.1738

[B43] Nagendra H. Kumar V. Mukherjee S. (2015). Research article cognitive behavior evaluation based on physiological parameters among young healthy subjects with yoga as intervention. Evid. Based Complement. Alternat. Med. 2015:821061. 10.1155/2015/821061 25759746 PMC4339827

[B44] Nath N. Kalatzis A. Stanley L. (2023). “Measuring user engagement in virtual, augmented, and mixed reality interventions for stress reduction,” in International Conference on Human-Computer Interaction (Cham: Springer Nature Switzerland), 570–583. 10.1007/978-3-031-48041-6_38

[B45] Nath N. Zavarelli J. Stanley L. Kalatzis A. Molina K. Lundberg C. Litwin A. (2024). “Integrating cognitive behavioral therapy and heart rate variability biofeedback in virtual reality, augmented reality, and mixed reality as a mental health intervention,” in 2024 IEEE Conference on Virtual Reality and 3D User Interfaces Abstracts and Workshops (VRW) (IEEE), 1200–1201. 10.1109/VRW62533.2024.00394

[B46] Olson I. R. McCoy D. Klobusicky E. Ross L. A. (2013). Social cognition and the anterior temporal lobes: a review and theoretical framework. Front. Hum. Neurosci. 7:404. 10.1093/scan/nss119 23051902 PMC3575728

[B47] Owens D. Mitchell A. Khazanchi D. Zigurs I. (2011). An empirical investigation of virtual world projects and metaverse technology capabilities. ACM SIGMIS Database 42, 74–101. 10.1145/1952712.1952717

[B48] Parameshwaran D. Thiagarajan T. C. (2019). Characterizing peaks in the EEG power spectrum. Biomed. Phys. Eng. Express 5:45023. 10.1088/2057-1976/ab29d0

[B49] Peshkovskaya A. Babkina T. Myagkov M. (2018). Social context reveals gender differences in cooperative behavior. J. Bioecon. 20, 213–225. 10.1007/s10818-018-9271-5

[B50] Peterson M. (ed.). (2015). The Prisoner's Dilemma. Cambridge, UK: Cambridge University Press. 10.1017/CBO9781107360174

[B51] Petruse R. E. Grecu V. Chiliban M. B. Tâlvan E. T. (2024). Comparative analysis of mixed reality and powerpoint in education: tailoring learning approaches to cognitive profiles. Sensors 24:5138. 10.3390/s24165138 39204835 PMC11360204

[B52] Poupard M. Larrue F. Sauzéon H. Tricot A. (2024). A systematic review of immersive technologies for education: learning performance, cognitive load and intrinsic motivation. Br. J. Educ. Technol. 56, 5–41. 10.1111/bjet.13503

[B53] Pushpa A. Shukla N. Horal L. Kivshyk O. Stepaniuk O. Reznik N. P. (2024). “Evolving horizons of work: unravelling the conceptual and future research dimensions of digital workspaces,” in AI in Business: Opportunities and Limitations: Volume 1, (Springer Nature Switzerland: Cham), 585–598. 10.1007/978-3-031-48479-7_50

[B54] Riva G. Di Lernia D. Sajno E. Wiederhold B. (2021). Virtual reality therapy in the metaverse: merging VR for the outside with VR for the inside. Annu. Rev. CyberTherapy Telemed. 19, 115–122.

[B55] Soni S. Overton E. Kam J. Pexman P. Prabhu S. Garza J. Saez I. Girgis F. (2024). Intracranial recordings reveal high-frequency activity in the human temporal-parietal cortex supporting non-literal language processing. Front. Neurosci. 17:1304031. 10.3389/fnins.2023.1304031 38260011 PMC10800947

[B56] Soroko N. V. Lytvynova S. H. (2021). “The benefits of using immersive technologies at general school,” in International Conference on Information and Communication Technologies in Education, Research, and Industrial Applications (Cham: Springer International Publishing), 247–257. 10.1007/978-3-031-14841-5_16

[B57] Souchet A. D. Lourdeaux D. Pagani A. Rebenitsch L. (2023). A narrative review of immersive virtual reality's ergonomics and risks at the workplace: cybersickness, visual fatigue, muscular fatigue, acute stress, and mental overload. Virt. Real. 27, 19–50. 10.1007/s10055-022-00672-0

[B58] Sung K. Dolcos S. Flor-Henry S. Zhou C.-C. Gasior C. Argo J.. (2011). Brain imaging investigation of the neural correlates of observing virtual social interactions. J. Vis. Exp. 6:e2379. 10.3791/2379 21775952 PMC3196171

[B59] Sweller J. (2011). “Cognitive load theory,” in Psychology of Learning and Motivation, eds. J. P. Mestre and B. H. Ross (San Diego, CA: Academic Press), 37–76. 10.1016/B978-0-12-387691-1.00002-8

[B60] Tomasi S. Schuff D. Turetken O. (2018). Understanding novelty: how task structure and tool familiarity moderate performance. Behav. Inf. Technol. 37, 406–418. 10.1080/0144929X.2018.1441325

[B61] Tremmel M. Schaefer M. Huster R. Fink G. Sch'´utz-Bosbach S. (2019). Modulation of cortical activity in 2D versus 3D virtual reality environments: an EEG study. NeuroImage 206:116311.31669411

[B62] Wang L. Zhang T. Guo M. Xu G. (2020). Eeg signal analysis of fatigue caused by virtual reality immersive visual experience. Chin. J. Biomed. Eng. 39, 160–169.

[B63] Weiss E. M. Kemmler G. Deisenhammer E. A. Fleischhacker W. W. Delazer M. (2003). Sex differences in cognitive functions. Pers. Individ. Differ. 35, 863–875. 10.1016/S0191-8869(02)00288-X

[B64] Wu L. Kane G. (2016). Network-Biased Technical Change: How Modern Digital Collaboration Tools Overcome Some Biases But Exacerbate Others. Labor: Human Capital eJournal.

[B65] Yaqob M. Hafez M. (2023). “Metaverse—an overview of daily usage and risks,” in Proceedings of the 2022 OPJU International Technology Conference on Emerging Technologies for Sustainable Development, OTCON 2022 (New York, NY: IEEE). 10.1109/OTCON56053.2023.10113922

