# Supplementary material for: Assessing the impact of virtual workplaces on collaboration and learning
Source: Front Psychol. 2025 Jun 19;16:1581029. doi: 10.3389/fpsyg.2025.1581029 (PMC12223796; doi:10.3389/fpsyg.2025.1581029)
Supplement: Supplementary file 1 [file Data_Sheet_1.pdf]

Supplementary Materials

S1. VR Experience Questions

1. **Have you ever used Virtual Reality headsets?**
   - Never
   - 1-2 times
   - 3-4 times
   - More than 5 times
   - I use them regularly
2. **Have you ever entered a 3D Metaverse platform, such as “Second Life,” “Decentraland,” “VRChat,” or others?**
   - Never
   - Yes, but only once to try
   - Yes, occasionally
   - Yes, I used it regularly in the past
   - Yes, I still use it regularly
3. **How often do you play online video games?**
   - Never played video games
   - Occasionally during the year
   - About once a month
   - About once a week
   - More than once a week
4. **Are you familiar with creating content or customizing avatars in the context of the Metaverse?**
   - Yes
   - No
5. **Have you ever participated in virtual events or conferences where you interacted with others through avatars or digital representations?**
   - Yes
   - No
6. **Have you ever participated in initiatives or programs involving Virtual Reality or the Metaverse, such as virtual art, immersive experiences, or virtual spaces dedicated to socialization?**
   - Yes
   - No

S2. Post-Presentation Test

1. **What is the approximate weight of the brain?**
   - 1,250g
   - 860g
   - 1kg
2. **Which of the following animals has the most neurons?**
   - Lion
   - Monkey
   - Elephant
3. **At what age is the maximum number of neurons reached?**
   - 30-40 years
   - 20-25 years
   - 50 years
4. **What is the name of the mechanism through which neurons communicate with each other?**
   - Connection
   - Interconnections
   - Synapse
5. **Which substances allow neurons to communicate with each other?**
   - Mineral salts
   - Neurotransmitters
   - Sugars
6. **What percentage of the human body weight is accounted for by the brain?**
   - 2%
   - 10%
   - 20%
7. **What is the most primitive region of the brain?**
   - The cerebellum
   - The brainstem
   - The sensory cortex
8. **How do the senses work?**
   - Each sense has a function and works in isolation

- Some senses "collaborate" with each other while others work in isolation
- The senses "collaborate" with each other

9. **What is the full name of patient HM?**
   - Homer Morris
   - Henry Molaison
   - Harry Mickelson

10. **In ancient times, where was it thought that emotions resided?**
    - Lungs
    - Heart
    - Brain
